# Tailoring Can Improve Consumers’ Hygienic Food-Handling Behavior to Reduce the Transmission Risk of Antimicrobial-Resistant Bacteria through Food

**DOI:** 10.3390/ejihpe12020014

**Published:** 2022-02-01

**Authors:** Claudia Freivogel, Sarah H. Lehmann, Vivianne H. M. Visschers

**Affiliations:** School of Applied Psychology, University of Applied Sciences and Arts Northwestern Switzerland, Riggenbachstrasse 16, 4600 Olten, Switzerland; sarahheather.lehmann@fhnw.ch (S.H.L.); vivianne.visschers@fhnw.ch (V.H.M.V.)

**Keywords:** tailored intervention, antimicrobial resistance, consumers, food-handling measures, hygienic behavior, randomized control trial

## Abstract

Antimicrobial-resistant (AMR) bacteria spread via food to humans and can seriously impair infection treatment. Hygienic food handling is an effective measure to avoid the transmission of bacteria. Therefore, we tested three types of interventions (tailored, generic, and no intervention) for their effectiveness in improving consumers’ hygienic food handling against the spread of antimicrobial-resistant bacteria through foods in a longitudinal randomized control trial. We based the determinants of hygienic food-handling behavior on the Health Action Process Approach (HAPA). The tailored intervention raised self-reported hygienic food handling, self-efficacy, and perceived likelihood of risk compared to no intervention. Moreover, interventions yielded different effects for participants with high vs. low intentions to improve their food-handling behavior. However, there were no differences between the tailored and generic interventions. More research is needed to find out whether including other behavior change techniques in the tailored intervention may increase the effect of tailoring.

## 1. Introduction

Hygienic food handling is regarded as an effective and simple measure in preventing foodborne infections [1]. These infections can be caused by antimicrobial-resistant (AMR) bacteria, which increase the burden of illness because treatment of infections with AMR bacteria is less effective, more costly, and more toxic than that of infections with bacteria not resistant to antibiotics [2,3]. Many consumers are unaware of their responsibility to prevent AMR as they do not see their behavior as potentially hazardous [4]. Consequently, they do not follow recommended hygienic food practices for cooking animal food products, e.g., [5,6], although raw meat, especially raw poultry, constitutes a risk for harboring AMR bacteria [7,8]. Consumers need to be aware of the transmission risk of AMR during food handling and understand that some habits must be changed when preparing food. Thus, an intervention is needed to foster the implementation of various hygienic food handling practices. The purpose of this study was to examine the effectiveness of a tailored online intervention compared with a nontailored online intervention and with no intervention in increasing hygienic food-handling behavior among consumers.

### 1.1. Tailored Intervention to Change Behavior

The aim of tailoring is to change behavior by offering personalized information and support. Personally relevant content is expected to lead to more behavioral change than a generic intervention (i.e., standardized information). There is evidence that behavior-change programs using computer tailoring are a successful strategy for health education [9,10,11,12,13]. The superior effects of a tailored intervention compared to a generic intervention were found for various behaviors (e.g., workplace sitting; fruit, vegetable, and fat intake; and also hygienic behavior such as hand hygiene) [14,15,16,17]. Little is known about the effectiveness of tailored interventions to promote hygienic food handling among consumers, let alone with regard to the reduction in the transmission risk of AMR bacteria. Therefore, research is required to investigate the effectiveness of tailoring on hygienic food-handling behavior. Previous research on generic interventions found that, despite an increase in hygienic behaviors, participants continued to use risky practices after the intervention, suggesting that the transmission risk of foodborne diseases remained [18]. A tailored intervention targeting empirically assessed attributes of the recipients that are relevant to the outcome behavior is expected to yield more behavioral change than a generic intervention.

However, certain variables that may moderate the effects of a tailored intervention on behavior change [12] are worth consideration. For example, interventions with more than one tailored message had greater effectiveness than those with only one. Additionally, the length of the first follow-up time point (ranging from one week to 18 months) correlated with the effect size, whereby studies with shorter follow-up periods (e.g., two weeks) had larger effects on health behavior than studies with longer follow-up periods (e.g., one year). Moreover, to increase the effectiveness of a tailored intervention, a theory-based approach is needed [19]. Interventions that made extensive use of theory tended to have larger effects on behavior than interventions that made less extensive or no use of theory [20]. In the study presented here, we therefore develop a theory-based online-delivered tailored intervention with three intervention points and four measurements and compare its effects on hygienic food-handling behavior to a generic intervention and no intervention.

### 1.2. Relevant Theoretical Determinants

Our intervention is based on the health action process approach (HAPA, [21]). The HAPA describes the determinants of health behavior that should thus be considered in behavioral intervention. It was successfully applied in predicting hygienic food handling to reduce the transmission risk of bacteria, such as AMR bacteria [22,23]. Moreover, a HAPA-based tailored hand hygiene intervention for professionals was shown to prevent infections with multidrug-resistant bacteria [16].

According to the HAPA, people undergo different phases while adopting a new behavior. Individuals in different phases face different problems and barriers and can thus be helped by different personalized types of intervention [24]. The HAPA specifies the factors responsible for three different phase transitions, supported by empirical research [25]. The preintentional phase includes individuals who did not yet set the goal to act. They are described as ‘nonintenders’. Research indicated that the determinants of the preintentional phase of the HAPA model are important predictors of hygienic food-handling behavior [23,26]. Additionally, self-efficacy is one of the theoretical constructs that showed the most consistent effects of being tailored to [12]. It describes an individual’s belief in his/her own capacity to execute hygienic food-handling measures. Risk perception and outcome expectancies are other relevant preintentional variables in the HAPA to tailor the intervention to [22]. Risk perception describes the perceived susceptibility (i.e., likelihood of being infected with AMR through food) and perceived severity (i.e., the seriousness of the health consequences of an AMR infection) of a health threat. Outcome expectancy is a person’s beliefs regarding positive or negative consequences due to a certain behavior.

People in the postintentional phase of HAPA have already formed intentions to handle food hygienically and want to carry out these intentions. They are considered ‘intenders’. For transitions from intention into action, planning was proposed as a key strategy, serving as a mediator between intentions and behavior [27]. Planning includes action planning, which pertains to thoughts of when, where, and how one intends to perform a behavior, and coping planning, which involves developing strategies for dealing with potential anticipated barriers. Studies about food handling revealed that coping planning predicts hygienic food-handling behavior, while action planning does not [22,23]. Action control is an additional self-regulatory determinant in the postintentional phase and describes the evaluation of one’s own behavior with regard to a behavioral standard.

After the postintentional phase, i.e., the action phase, the newly adopted behavior should be controlled by cognition and regulated actively to yield a long-term behavior change [28]. If a behavior is performed repeatedly, then its execution becomes gradually automated, and the habit strength increases. Previous research suggests that habits are formed by repeating a behavior over time in a consistent context or in response to a cue [29]. Therefore, it is important to build hygienic food-handling practices as habits. An intervention with habit-formation approaches was an effective strategy to increase habit strength and food-handling behavior over four weeks [30,31].

### 1.3. The Present Study

Our overall aim was to develop an evidence-based intervention to enhance hygienic food-handling behavior in the long term to mitigate the AMR transmission risk among consumers. A secondary aim was to reveal if providing tailored information would yield a superior effect to providing generic information. We thus developed an intention-based, computer-delivered, and tailored intervention based on the HAPA model to promote hygienic food handling and reduce the transmission risk of AMR. To our knowledge, no prior study used this approach. We hypothesized that nonintenders would mainly benefit from an intervention targeting the preintentional factors of self-efficacy, risk perception, and outcome expectancies to increase intention for hygienic food handling, whereas an intervention targeting the postintentional determinants of coping planning, action control, and habit formation would be beneficial in promoting hygienic food handling among intenders. The tailored intervention incorporated three different behavior change techniques (BCTs) at three time points. The effect on a HAPA determinant was expected after receiving the BCT targeting that determinant. Therefore, hypotheses vary with respect to the time point of the expected effect. Before data collection, we preregistered our study with the following hypotheses on the Open Science Framework (https://osf.io/3ru4x, accessed on 30 January 2022):

1. Participants in the tailored intervention condition show higher levels of hygienic food-handling behavior, intention, self-efficacy, and risk perception at T2, T3, and T4, compared to that of participants in the generic intervention condition and the control condition.
2. Participants in the tailored intervention show lower levels of negative outcome expectancies at T3 and T4, compared to that of participants in the generic intervention and the control condition.
3. Participants in the tailored intervention show higher levels of coping planning and action control at T3 and T4, compared to that of participants in the generic intervention and the control condition.
4. Hygienic food-handling behavior increases over time among participants in the tailored and generic interventions, whereas this behavior remains at the same level from T1 to T4 in the control group.
5. The increase in hygienic food-handling behavior over time is higher for participants in the tailored intervention than in the generic intervention.

## 2. Materials and Methods

### 2.1. Procedure and Study Design

**Figure 1:**
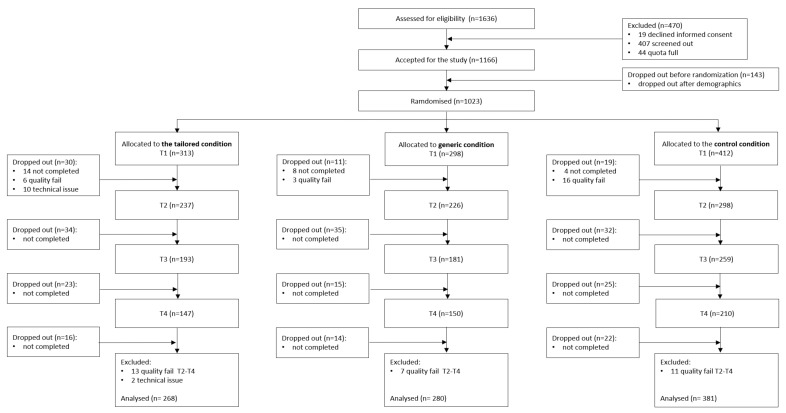
Flowchart of participants per condition and measurement, including dropouts and reasons.

Between May and July 2020, we conducted an intervention study with four repeated measures to assess the relative efficacy of a tailored intervention and a generic intervention compared with that of a control condition. The questionnaires and interventions were programmed in Questback [32]. All participants were provided with a brief statement explaining the procedure and purpose of the study. After participants provided informed consent and they completed a baseline survey before being assigned to one of three conditions. We expected a higher dropout rate in the control condition because the participants in this condition did not receive any BCT after completing the questionnaire at T1, T2, and T3, in contrast to those in the tailored and generic conditions. Hence, 40% of participants were randomly assigned to the control condition and 30% each to the generic and tailored intervention. Only participants who correctly completed the quality fail question at the baseline (T1) survey were invited to the survey at the second measurement (T2) approximately two weeks later. Again, two weeks after finishing the T2 survey, participants were prompted by email to take the survey for the third measurement (T3). One month after T3, participants were invited by mail to participate in the follow-up survey (T4). The participants received credits for each completed questionnaire (redeemable points equivalent to 0.55 EUR at T1 and T3; 0.90 EUR at T2 and T4). After finishing the T4 survey, participants were debriefed received additional credits (redeemable points equivalent to 1.50 EUR). Figure 1 illustrates the participant flowchart.

### 2.2. Measures

At each time point, participants completed a questionnaire before receiving a BCT (except for those in the control condition, who only completed the questionnaire). The questionnaire was formed by the existing self-reported health behavior and HAPA literature (see Supplementary Materials, Table S1). Items to assess hygienic food handling to prevent the spread of AMR were adapted from previous studies as there were no measures available [22,23].

All four surveys measured self-reported hygienic food-handling behavior, the variables of the HAPA model, and habitual hygienic behavior. Self-reported hygienic food-handling behavior was not assessed if a person did not prepare raw meat between two measurement time points. It included questions regarding the frequency of implementing the hygienic measures and was assessed with six items, which were rated on six-point response scales ranging from ‘never’ to ‘always’. In the questionnaires at T2, T3, and T4, participants were asked how often they prepared raw meat, poultry, fish, or seafood since the previous measurement time point. At T1, additionally, AMR knowledge was measured with six items (response options: ‘true’, ‘false’, ‘I do not know’) after receiving the intervention. Incorrect and ‘I do not know’ answers were merged together to conveniently sum up the amount of correct knowledge per respondent (ranging between 0 and 6). The main goal of the knowledge assessment was to determine whether participants watched the video carefully (see Supplementary Materials, Table S2).

The following measures incorporated determinants of the preintentional and postintentional phases of the HAPA model and were assessed at all four time points. The preintentional components included self-efficacy, outcome expectations, risk perception, and intention. Risk perception was assessed using six items, whereby three items each measured the likelihood and the severity of an AMR infection through food handling. Participants responded on seven-point scales ranging from ‘unlikely’ to ‘very likely’. Self-efficacy was measured by six questions. Participants responded on seven-point scales ranging from ‘not at all confident’ to ‘very confident’. Outcome expectancies were assessed with eight items. The five items of negative outcome expectancies were designed to measure prevalent beliefs regarding the cons of hygienic food handling. The three items of positive outcome expectancies measured participants’ beliefs regarding the antibacterial effect of food-handling measures. Both positive and negative outcome expectancies were measured with seven-point scales ranging from ‘do not agree at all’ to ‘fully agree’. Behavioral intentions to implement the food handling practices were measured by six questions with seven-point response scales ranging from ‘do not intend at all’ to ‘fully intend’. At baseline, intention to adopt a food-handling measure was not assessed if a person indicated that they had already implemented it.

The postintentional phase components included coping planning, action control and habit formation. Coping planning was measured by four questions. Individuals were asked how they plan to overcome barriers that prevent them from implementing hygiene measures. The seven-point response scales ranged from ‘do not agree at all’ to ‘fully agree’. Action control was measured with two items on seven-point response scales ranging from ‘do not agree at all’ to ‘fully agree’. Habitual behavior was measured with four items from the self-report habit index (SRHI) relating to automaticity [33].

At T4, participants in the tailored and generic intervention conditions were further asked to evaluate the information received on 12 six-point semantic differential scales with the following adjectives: informative, alarming, convincing, motivating, understandable, realistic, important, sufficient, relevant, practicable, effortful, and realistic. Moreover, AMR knowledge was measured again with the same six items as in the baseline assessment.

To identify random answers, we integrated a control item (e.g., ‘This is a control question. Please select ‘2’ on the scale’) at every measurement time point. Participants’ gender, birth year, education level, household composition, health state, and experience with AMR were assessed at T1.

### 2.3. Intervention Descriptions

BCT taxonomies were used to identify possible strategies to increase hygienic food-handling behavior [34,35,36]. We selected BCTs that are most appropriate for laypeople and an online intervention [37].

Participants in the control condition received no intervention. Only a definition of AMR and the relationship to food was presented to participants at T1 (see Supplementary Materials S1).

Participants in the generic intervention condition received an educational video at T1 about food as an AMR exposure source that was proven to increase AMR knowledge and risk perception [38]. It included risk information and the four core food safety practices: ‘clean, chill, cook and separate’ [1]. The video addressed common concerns and misconceptions, such as the frequently mentioned belief that organic food products are not contaminated with AMR bacteria [4]. At T2 and T3, participants received a reminder to implement measures regarding clean, chill, cook, and separate. These measures were illustrated and accompanied by a short text (see Supplementary Materials S2) [39].

The tailored intervention started with the same educational video as the generic intervention at T1. Afterwards, participants received tailored information that focused on improving an individual’s hygienic food-handling behavior through reinforcing preintentional or postintentional predictors (see Supplementary Materials, Figure S1). On the basis of their assessments, participants were categorized as nonintenders or intenders of hygienic food-handling behavior. Individuals who indicated high intention (intention response = 7) to change at least two food-handling practices that they did not yet implement regularly (response of self-reported behavior < 6) were considered as intenders, whereas the other participants were classified as nonintenders.

BCTs for nonintenders in the tailored intervention included triggering perceived self-efficacy by providing feedback on participants’ past performance and comparing participants’ performance with the performance of others at T1 [40], decisional balance by mitigating perceived negative outcome expectancy (‘cons’) at T2 [41] and personalized risk messages to increase perceived risk perception at T3 (see Supplementary Materials S3 for the personalized risk message). Since self-efficacy is the strongest predictor of intention to handle food hygienically, self-efficacy was targeted at T1 [22,23].

BCTs for intenders in the tailored intervention targeted behavioral regulation and included goal setting by prompting participants to choose which two hygienic measures they wanted to adopt because self-guided behavior change is a common form of long-term behavior change [41,42]. Setting measures was accompanied with instructions on how to perform hygienic practices to trigger beliefs about capabilities and to avoid repression of health concerns, which is a tactic to cope with a health threat (see Supplementary Materials S3) [43,44]. In a previous study, goal setting successfully increased the planning and execution of hygienic food handling that reduces the transmission risk of AMR [38]. Goal setting also leads to an increase in motivation, which in turn can increase behavior [45]. At T2 and T3, intenders were additionally divided into ‘responders’ and ‘nonresponders’ depending on whether they continued with their intended goals during the past two weeks. Responders were those who implemented both selected goals consistently, while nonresponders implemented at least one measure inconsistently. The BCT directed at responders was habit formation to develop a habit of implementing hygienic measures without consciously remembering [46]. Participants were encouraged to identify situational, contextual, or time-based cues that could prompt them to implement the chosen hygienic, safe food-handling measures. BCTs for nonresponders included stimulating coping plans by anticipating existing and potential barriers. If a participant remained a nonresponder at T3, the BCT coping advice was applied, whereby suggestions were given on how to cope with personally relevant barriers [47].

### 2.4. Participants

Participants were recruited from an online survey panel operated by an internet marketing and research institute. Eligible consumers prepared raw meat, poultry, fish, or seafood regularly, did not already adopt all the measured food-handling practices, were living in Switzerland, 18 years or older, and proficient in German. At T1, quotas were set for gender (50% female; 50% male) and age (33% 18–40 yrs.; 42% 41–60 yrs.; 25% 61–100 yrs) to ensure that the sample was representative of the Swiss adult population [48]. Power calculations for a 3 (between-subjects) x 4 (within-subjects) MANOVA using G*Power indicated that a sample size of 467 at T4 was required to detect an effect size of *f* = 0.15, based on previous related research (e.g. [49]). Studies published in the field of tailored internet-based health interventions usually report small to moderate effect sizes, depending on the targeted behavior. We a priori calculated the required sample size for the repeated-measures MANOVA because most of the sample size methods for multilevel studies are based on approximations and make simple assumptions about the study design, and are therefore recommended with caution [50]. To achieve a statistical power of 95% and an expected response rate of 80%, we aimed to allocate about 1000 subjects to one of the three conditions at T1.

Table 1 displays the demographic characteristics of the participants in each condition. After data cleaning, the final study sample consisted of 929 respondents (28.8% in the tailored, 30.2% in the generic, and 41.0% in the control conditions) who completed the baseline survey, 614 participated at T2, 518 participated at T3, and 398 (26.6% tailored, 30.7% generic, 42.7% control) participated in the follow-up survey at T4. The follow-up rate was 79.0% between T1 and T2, 83.2% between T2 and T3, and 80.1% between T3 and T4.

### 2.5. Statistical Analyses

SPSS 25 [51] was used for data cleaning and all analyses, except Mokken scale analysis (MSA) and multilevel modeling (MLM), which were performed using R (Version 4.0.2) [52]. As preliminary data analyses, we applied scale validation analyses [53]. We conducted MSA with the HAPA variables using the *mokken* package (Version 3.0.3) in R. [54,55]. The *mice* package (Version 3.12.0) was applied to impute missing data. The *aisp* function in the *mokken* package was used to identify potential Mokken or unidimensional scales. The individual item scalability coefficients (*H_i_*) ranged from −0.14 to 0.33, and thus most items were below the accepted cut-off of 0.30 [56]. One positive outcome expectancy item (POE1) was challenging and thus excluded from the following analyses [56]. We kept one discerning self-reported behavior item (B4) because various preventive measures were assessed, and therefore some heterogeneity was to be expected. A challenging negative outcome expectancy item (NOE3) was retained because it provides a useful indicator of the negative expectations of engaging in hygienic food handling [57]. Next, exploratory factor analysis (EFA) was performed on the remaining HAPA items. The EFA suggested to split risk perception into two constructs: likelihood and severity, which is in line with theory [58]. Items assessing self-reported behavior were divided over two factors, which is not surprising because they cover various measures. The intention items were split over the same two factors. Cronbach’s alpha was then calculated to test each construct’s internal consistency (behavior: α = 0.53, *N* = 6; intention: α = 0.89, *N* = 6, pre- and postintentional variables: 0.76 < αs < 0.93, 2 < *N*s < 6).

Dropout analyses were conducted using chi-square test for gender, education level, and household composition, and a multivariate analysis of variance (MANOVA) with planned contrasts for age and the HAPA variables (see Supplementary Materials, Table S3). Moreover, the chi-square test was applied to investigate dropout rates between conditions.

To check whether there were differences among the final sample between the three conditions on sociodemographic variables, one-way analyses of variance (ANOVAs) were conducted on age and food preparation frequencies and a chi-square test on remaining demographics (Table 1). We investigated whether there were differences between the three conditions regarding HAPA variables at baseline using one-way MANOVA.

MLM was applied to examine the differences between conditions and across time (T1 to T4) on the HAPA determinants for each participant. We used the *lme* function in the R package *nlme* [59]. MLM partitions the residual variance into a between-participant component and a within-participant component, which allows modeling the variability in regression slopes explicitly. Moreover, MLM does not require complete data sets, so that when data are missing for one time point, they do not need to be imputed, nor does the whole case need to be deleted. An MLM was fitted using the maximum likelihood estimation for each dependent variable (i.e., ten analyses in total). Repeated measures (level 1) were nested within participants (level 2). The intraclass correlations (ICC) were calculated as the ratio of the random intercept variance (between-person) to the total variance, defined as the sum of the random intercept variance and residual variance (between- and within-person). First, we assessed the need for MLM by ascertaining whether variation exists across individuals. We started testing simpler models including only the intercept and added the random effects stepwise. A smaller akaike information criterion (AIC) thereby indicates a better fitting model. To model the covariance structure, we used the *corCar(1)* function because time points were not equally spaced [60]. Intervention conditions were included into the model as two dummy-coded variables with tailored intervention as the reference group [61]. Cross-level interactions between time effects and intervention conditions were inserted to examine how intervention affected individual growth trajectories. Moreover, we added each individual’s HAPA phase at each time point as a predictor to the model. This dichotomous dummy variable indicated whether a participant was an intender or not (i.e., still in the preintentional phase). Once participants became an intender, they remained intender and received the intender intervention until T4. Including the three-way interaction between time, intervention and HAPA phase enabled us to assess whether the tailored intervention was more effective among intenders or nonintenders over time. To break down a significant interaction, we reran the analysis separately for the relevant conditions with the time and phase variables as predictors [60]. The R *multcomp* package was used for posthoc testing of significant interactions with the time variable. To verify whether a nonlinear pattern of growth across time applied to our data, the best-fitting function of time was selected—namely, time effects with quadratic (time2) or even cubic (time3) trends [60]. All reported *p*-values were evaluated using a significance level of α = 0.05.

The effect of the intervention on knowledge was investigated with a repeated-measures ANOVA as knowledge was only assessed at T1 and T4. The between-groups factor was conditions (tailored vs. generic vs. control), and the within-subjects factor was time point (T1 vs. T4). Assumptions for a 3 × 2 mixed repeated-measures ANOVA were verified. The significance level was set at α = 0.025 because the homogeneity of variances and variance–covariance matrices were violated [62]. Last, an independent *t*-test was performed to test for differences in evaluating the information between the tailored and generic conditions at T4.

## 3. Results

### 3.1. Sample

At T1, respondents who did not complete the questionnaire or did not correctly respond to the control question were excluded. We found no outliers at T1 based on a three SD rule. Only participants who completed all items at one time point were invited for the following questionnaire. From the remaining data set at T4, we excluded participants who did not correctly respond to the control question at T2 to T4 (see Figure 1). From the participants who were excluded from the final dataset, the majority dropped out before randomization (see Figure 1). Of those who reached randomization but were excluded from the final dataset, 45 (47.9%) were in the tailored condition, 18 (19.1%) in the generic condition and 31 (33.0%) in the control condition. Dropout analyses showed that the percentage of participants who dropped out after T1 or T2 and those who completed all four questionnaires differed by age and household composition, but not by gender or education (see Supplementary Materials, Table S3). There were no differences between dropouts and completers concerning the postintentional HAPA variables at T1 and most preintentional HAPA variables at T1. The perceived likelihood of risk at T1 was higher among participants who dropped out after T2 than participants who completed all four questionnaires.

The demographic sample characteristics for each condition are presented in Table 1 (see Supplementary Materials, Table S4 for the total sample characteristics). Among the final sample, demographic characteristics (i.e., gender, age, education level and household composition) did not differ among conditions, which indicated that the randomization was successful (see Table 1). The *p*-value for age was marginally insignificant, but age did not significantly correlate with the dependent HAPA variables’ negative outcome expectancy and both risk-perception scales (*r*_s_s < 0.06, *p*s > 0.06). The correlations with age and self-reported behavior (*r*_s_ = 0.15, *p* = 0.001), intention (*r*_s_ = 0.10, *p* = 0.003), self-efficacy (*r*_s_ = 0.16, *p* = 0.001), positive outcome expectancy (*r*_s_ = 0.11, *p* = 0.001), coping planning (*r*_s_ = 0.10, *p* = 0.004), action control (*r*_s_ = 0.15, *p* = 0.001) and habit (*r*_s_ = 0.10, *p* = 0.002) were significant but very weak. Therefore, we did not control for age in the further analyses. Raw meat, poultry and fish were prepared with equal frequency across the three conditions.

### 3.2. HAPA Variables at Baseline

The one-way MANOVA found no significant multivariate effect of conditions on the HAPA variables at T1, *F*(20, 1836) = 0.85, *p* = 0.66, indicating that no differences between the conditions existed before receiving the intervention. Participants’ intention and self-efficacy were already high at baseline, whereas perceived likelihood was the lowest. Overall, participants reported handling their food rather hygienically. Spearman correlations between the HAPA variables were according to theory. The relationship between self-reported behavior and intention (*r_s_* = 0.52, *p* = 0.001) and behavior and the postintentional variables were moderate to strong (0.36 < *r_s_*s < 0.59, *p*s < 0.001), whereas the relationships between behavior and the preintentional variables were weak (0.07 < *r_s_* < 0.28, *p*s < 0.03). Only the relationship between behavior and the preintentional variable self-efficacy was strong (*r_s_* = 0.44, *p* = 0.001), which is also in accordance with the theory (see Supplementary Materials, Table S3 for descriptive statistics of the HAPA variables).

### 3.3. MLM

#### 3.3.1. Model Fit

The ICC indicated that 69% of the variance in self-efficacy, 56% in likelihood, 53% in severity, 48% in positive outcome expectancy, 67% in negative outcome expectancy, 64% in intention, 53% in coping planning, 63% in action control, 69% in habit, and 59% in behavior was between participants. The random intercept and random slope model yielded a better fit for self-efficacy, likelihood, intention, coping planning, action control, habit and behavior than that of the fixed effects model and the random intercept model, meaning that, for example for food handling, not only the starting point at T1 (intercept) but also the change over time (slope) differed between participants.

The random intercept model yielded a better fit for severity, positive outcome expectancy and negative outcome expectancy than the fixed effects model or the random intercept and random slope model, meaning that, e.g., perceived severity of risk differed at T1 between participants (intercept), but the change in perceived severity over time (slope) did not. Correlations of the intercepts and slopes are reported for random intercept and random slope models. The correction for first-order autocorrelation with a continuous-time covariate structure significantly improved the model fit for likelihood (φ = −0.15) and action control (φ = 0.17). A positive correlation coefficient means that those with higher intercepts are associated with increased time trajectories, which is the course of a measured variable over time, whereas a negative coefficient means that those with lower intercepts are associated with increased time trajectories.

#### 3.3.2. Preintentional Determinants

##### Self-Efficacy

As shown in Table 2, we found neither a time effect nor an interaction effect between time and condition on self-efficacy. However, the three-way interaction between time, intervention and HAPA phase indicated that slopes varied between the tailored and control conditions. The interaction was broken down by conducting separate multilevel models on the tailored condition and the control condition. These analyses showed that for participants in the tailored condition, the HAPA phase, *b* = 0.62, *t*(429) = 6.33, *p* = 0.001, and the interaction between time and HAPA phase, *b* = –0.14, *t*(429) = –2.41, *p* = 0.02, significantly predicted self-efficacy, whereas time, *b* = 0.08, *t*(429) = 1.84, *p* = 0.07, did not. A multiple comparison showed a significant difference in self-efficacy between intenders and nonintenders at T1, *b* = 0.68, *z* = 6.39, *p* = 0.001, and T2, *b* = 0.40, *z* = 3.47, *p* = 0.01, but not anymore at T3 or T4, *b*s < 0.35, *z*s < 2.04, *p*s > 0.99. The negative gradient indicates that self-efficacy increased less among intenders than among nonintenders. For those in the control condition, the HAPA phase, *b* = 0.37, *t*(638) = 4.56, *p* = 0.001, significantly predicted self-efficacy, whereas time, *b* = −0.05, *t*(638) = −1.46, *p* = 0.14, and the interaction between time and the HAPA phase, *b* = 0.08, *t*(638) = 1.86, *p* = 0.06, did not. Hence, we found no superior effect of the tailored condition compared to the generic condition. Nevertheless, these results suggest that the tailored intervention was able to increase nonintenders’ self-efficacy, whereas self-efficacy did not increase among participants in the control condition. Additionally, the tailored intervention reduced the gap between nonintenders’ and intenders’ self-efficacy.

##### Risk Perception

The results showed that the perceived likelihood of risk increased over time (Table 2). There was no significant quadratic or cubic time effect. Moreover, the significant interaction effect of time and condition reflects the difference in slopes for time as a predictor of perceived likelihood of risk in the control condition compared to that of the tailored condition. That is, the perceived likelihood increased less among participants in the control condition compared to the tailored condition. Multiple comparisons showed no significant effects between conditions at a time point or between times points, *b*s < 0.31, *z*s < 2.79, *p*s > 0.11. We again found no superior effect of the tailored condition compared to the generic condition regarding likelihood compared to that of the control condition.

As shown in Table 2, we found neither a time effect nor an interaction effect between time and condition on the perceived severity of risk. Intenders perceived the risk as more severe than nonintenders. Although not significant, the negative gradient of time indicates that perceived severity decreased over time rather than increased.

##### Positive and Negative Outcome Expectancy

Positive outcome expectancy did not increase over time, nor did it differ between the conditions (Table 2). Intenders again showed more positive outcome expectancy than nonintenders.

The MLM on negative outcome expectancy showed no significant time effect or interaction effect between time and condition (see Table 2). However, the three-way interaction between time, intervention and HAPA phase indicated that slopes varied between the tailored and control conditions and between the tailored and generic conditions. Conducting separate multilevel models on each condition with time and HAPA phase as predictors showed that the HAPA phase, *b* = −0.37, *t*(429) = −3.39, *p* = 0.001, significantly predicted negative outcome expectancy in the tailored condition, but the time effect was not significant, *b* = −0.08, *t*(429) = −1.82, *p* = 0.07. There was neither a significant time effect in the generic, *b* = −0.02, *t*(454) = −0.36, *p* = 0.72, or control condition, *b* = 0.03, *t*(638) = 0.82, *p* = 0.41, and no significant HAPA phase effect in the generic, *b* = −0.18, *t*(454) = −1.58, *p* = 0.12, or control condition, *b* = −0.10, *t*(638) = −1.10, *p* = 0.27. Moreover, we found a significant interaction of time and HAPA phase for participants in the tailored condition, *b* = 0.20, *t*(429) = 3.39, *p* = 0.001, but not in the generic condition, *b* = −0.06, *t*(454) = −1.06, *p* = 0.29, or control condition, *b* = −0.06, *t*(638) = −1.33, *p* = 0.18. The positive gradient indicated that negative outcome expectancy increased more over time among intenders than nonintenders. Multiple comparisons showed that among participants in the tailored condition, intenders’ negative outcome expectancy significantly increased from T2 to T4, *b* = 0.40, *z* = 3.52, *p* = 0.01, whereas it did not change among nonintenders, *b* = −0.19, *z* = −1.17, *p* = 1.00.

##### Summary of Preintentional Variables

In sum, the results differed among the preintentional variables. The tailored intervention yielded a superior effect on self-efficacy and likelihood of risk compared to that of the control condition. However, we found no difference between the tailored and the generic intervention on the preintentional variables. Thus, the first hypothesis could only be partially confirmed. Positive outcome expectancy and perceived severity of risk did not change over time, both irrespective of the condition. Therefore, hypothesis one has to be declined with respect to severity. Negative outcome expectancy was the only variable for which a decrease instead of an increase over time was expected. Since a significant decrease was not achieved in any of the conditions, we could not confirm hypothesis two.

#### 3.3.3. Intention

As shown in Table 3, the results showed a significant increase over time in intention in all three conditions. Including a quadratic and cubic time effect significantly improved the model fit, suggesting a nonlinear pattern over time. We found no interaction effect between time and condition. The significant three-way interaction between time, intervention, and HAPA phase indicated that slopes varied between the tailored and control conditions.

**Figure 2:**
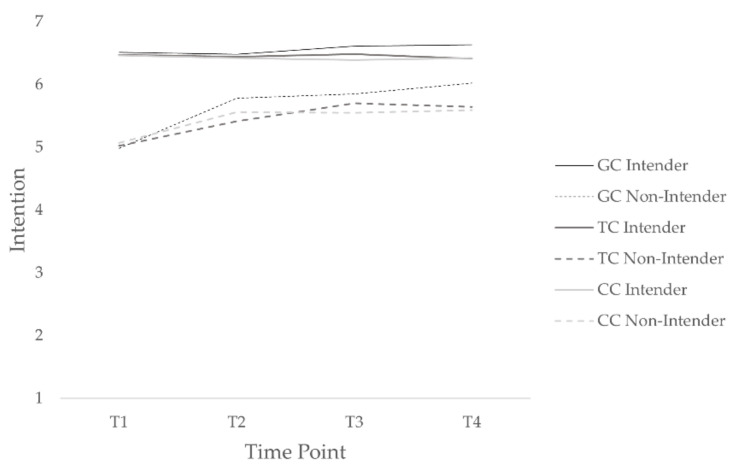
Intention among intenders and nonintenders in tailored, control, and generic conditions over time. GC denotes generic condition; TC: tailored condition; and CC: control condition.

This interaction was broken down by conducting separate MLM on the tailored condition and the control condition. These analyses showed a quadratic time effect for the participants in the control condition, but not for the participants in the tailored condition. Among participants in the tailored condition, time, *b* = 0.41, *t*(428) = 4.95, *p* = 0.001, the HAPA phase, *b* = 1.31, *t*(428) = 12.46, *p* = 0.001, and the interaction between time and HAPA phase, *b* = –0.34, *t*(428) = –5.53, *p* = 0.001, significantly predicted intention (see Figure 2). In the tailored condition, multiple comparisons showed that for nonintenders, a significant improvement in intention occurred from T1 to T2, *b* = 0.46, *z* = 4.95, *p* = 0.001, from T1 to T3, *b* = 0.70, *z* = 6.08, *p* = 0.001, and from T1 to T4, *b* = 0.72, *z* = 4.47, *p* = 0.001, but not for intenders, *b*s < −0.18, *z*s < −1.55, *p*s > 0.99. Although nonintenders’ intention increased over time, there was still a significant difference in intention between intenders and nonintenders at T1, *b* = 1.38, *z* = 12.18, *p* = 0.001, at T2, *b* = 0.87, *z* = 7.02, *p* = 0.001, at T3, *b* = 0.53, *z* = 3.77, *p* = 0.01, but not anymore at T4, *b* = 0.47, *z* = 2.57, *p* = 0.28.

For those in the control condition, time, *b* = 0.34, *t*(637) = 5.27, *p* = 0.001, the HAPA phase, *b* = −1.06, *t*(637) = 12.93, *p* = 0.001, and their interaction, *b* = −0.20, *t*(637) = −4.49, *p* = 0.001, significantly predicted intention. In the control condition, multiple comparisons showed that for nonintenders, a significant improvement in intention occurred from T1 to T2, *b* = 0.51, *z* = 6.80, *p* = 0.001, from T1 to T3, *b* = 0.49, *z* = 5.74, *p* = 0.001, and from T1 to T4, *b* = 0.53, *z* = 4.75, *p* = 0.001, but not for intenders, *b*s < −0.06, *z*s < −0.64, *p*s > 0.99. Although nonintenders’ intention increased over time, there was still a significant difference in intention between intenders and nonintenders at T1, *b* = 1.17, *z* = 13.31, *p* = 0.001, at T2, *b* = 0.61, *z* = 6.21, *p* = 0.001, at T3, *b* = 0.64, *z* = 6.02, *p* = 0.001, and at T4, *b* = 0.59, *z* = 4.56, *p* = 0.001.

The results showed no superior effect of the tailored condition on intention compared to the generic or control condition. Hence, hypothesis one could not be confirmed regarding intention. However, we found that in the tailored and control conditions, intention increased among nonintenders but not among intenders. The gap between intenders’ and nonintenders’ intentions disappeared over time in the tailored condition, whereas it remained in the control condition.

#### 3.3.4. Postintentional Determinants

##### Coping Planning

We found a significant time effect on coping planning (Table 3). Adding the quadratic and the cubic time effect significantly improved the model fit. There was no interaction effect between time and condition, but a three-way interaction between time, intervention and HAPA phase indicated that slopes varied between the tailored and control conditions. Breaking down the interaction showed no quadratic or cubic time effect for the tailored condition but did show a cubic effect for the control condition. In the tailored condition, time, *b* = 0.18, *t*(429) = 2.57, *p* = 0.01, the HAPA phase, *b* = 0.72, *t*(429) = 4.84, *p* = 0.001, and the interaction between time and the HAPA phase, *b* = −0.19, *t*(429) = −2.15, *p* = 0.03, significantly predicted coping planning. The negative gradient showed that coping planning increased less over time among intenders than among nonintenders. A multiple comparison revealed a difference in coping planning between nonintenders and intenders at T1, *b* = 0.82, *z* = 5.05, *p* = 0.001, but not anymore at T2, T3 or T4, *b*s < 0.42, *z*s < 2.01, *p*s > 0.99. In the control condition, time, *b* = 0.55, *t*(636) = 2.07, *p* = 0.04, and the HAPA phase, *b* = 0.27, *t*(636) = 2.09, *p* = 0.04, significantly predicted coping planning, whereas their interaction did not, *b* = 0.02, *t*(636) = 0.21, *p* = 0.83. Since coping planning had also increased in the control condition, the tailored condition did not yield a superior effect. Additionally, we found no difference between the tailored and generic interventions on coping planning.

##### Action Control

We found no time effect and no interaction effect between time and condition on action control (Table 3). Breaking down the significant three-way interaction showed no quadratic or cubic time effect for the tailored condition but did show a cubic effect for the control condition. In the tailored condition, time, *b* = 0.20, *t*(429) = 3.07, *p* = 0.001, the HAPA phase, *b* = 0.20, *t*(429) = 3.07, *p* = 0.001 and the interaction between time and the HAPA phase, *b* = −0.19, *t*(429) = −2.19, *p* = 0.03, significantly predicted action control. A multiple comparison revealed a difference in action control between nonintenders and intenders at T1, *b* = 0.70, *z* = 4.33, *p* = 0.001, but not anymore at T2, T3 or T4, *b*s < 0.52, *z*s < 2.90, *p*s > 0.10. In the control condition, time, *b* = −0.57, *t*(636) = −2. 47, *p* = 0.01, and the HAPA phase, *b* = 0.38, *t*(636) = 3.03, *p* = 0.001, significantly predicted action control, whereas the interaction between time and HAPA phase did not, *b* = 0.02, *t*(636) = 0.34, *p* = 0.73. Action control increased in the tailored and the control conditions, whereas we found a difference between the intenders and nonintenders in the tailored but not in the control condition. The results yielded neither a superior effect of the tailored intervention on action control compared to the generic intervention nor the control condition.

##### Habitual Behavior

We found neither an increase in habitual food handling over time nor a difference between conditions (Table 3). As to be expected from theory, intenders again showed more habitual behavior than nonintenders.

##### Summary of Postintentional Variables

Overall, we found less impact of the tailored intervention on the postintentional variables among intenders than on the preintentional variables among nonintenders. We could not prove the tailored intervention to be more successful in increasing the targeted postintentional determinants than the generic intervention or the control condition. Thus, hypothesis three had to be rejected. Although in the tailored intervention, intenders received a BCT to improve coping planning and the nonintenders did not, the latter group showed a higher level of coping planning than intenders. This closed the gap between the coping planning of intenders and nonintenders. The same accounts for action control.

#### 3.3.5. Self-Reported Behavior

As shown in Table 3, hygienic food-handling behavior increased significantly over time in all three conditions. The quadratic time effect was significant, suggesting that participants experienced a significant initial increase in hygienic food handling, which again decreased over time. Contrary to our hypotheses, neither condition nor the interaction effect between condition and time was significantly related to hygienic food handling. However, the three-way interaction between time, intervention and HAPA phase (being an intender or not) indicated that slopes varied between the tailored and control conditions. This interaction was broken down by conducting separate MLM on the tailored condition and the control condition. The models specified were the same as the main model but excluded the main effect and interaction term involving condition. These analyses showed a quadratic time effect for the participants in the control condition but not for the participants in the tailored condition.

**Figure 3:**
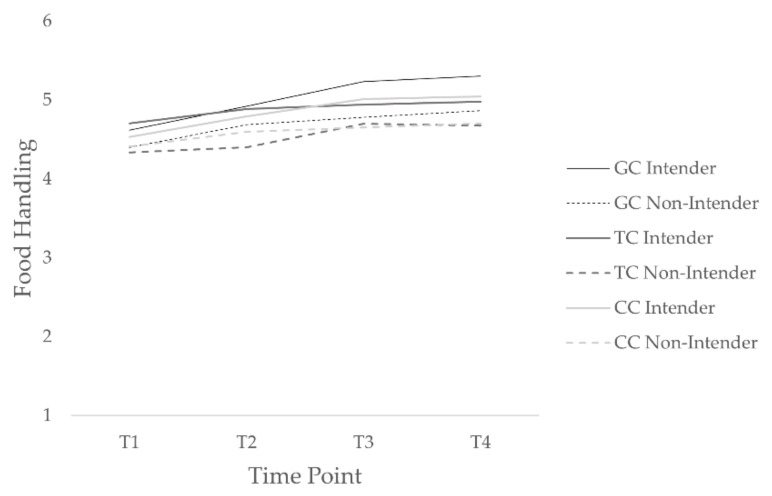
Increase in hygienic food-handling behavior among intenders and nonintenders in tailored, control, and generic conditions over time. GC denotes generic condition; TC: tailored condition; and CC: control condition.

That is, among participants in the tailored condition, time, *b* = 0.19, *t*(415) = 4.38, *p* = 0.001, and the HAPA phase, *b* = 0.23, *t*(415) = 2.69, *p* = 0.01, significantly predicted hygienic food handling, but the time and HAPA phase interaction did not, *b* = –0.09, *t*(415) = –1.71, *p* = 0.09. For those in the control condition, time, *b* = 0.21, *t*(617) = 3.63, *p* = 0.001, and the interaction between time and HAPA phase, *b* = 0.12, *t*(617) = 3.06, *p* = 0.001, significantly predicted hygienic food handling, but the HAPA phase did not, *b* = −0.13, *t*(617) = −1.85, *p* = 0.06. The interaction effect thus reflects the difference in slopes for time as a predictor of food handling in those who are intenders and those who are nonintenders. In the control condition, multiple comparisons showed that intenders’ hygienic food-handling behavior significantly improved from T1 to T2, *b* = 0.26, *z* = 3.60, *p* = 0.01, from T1 to T3, *b* = 0.49, *z* = 6.53, *p* = 0.001, from T1 to T4, *b* = 0.53, *z* = 6.72, *p* = 0.001, from T2 to T4, *b* = 0.26, *z* = 3.61, *p* = 0.01, and from T2 to T4, *b* = 0.23, *z* = 3.24, *p* = 0.03, whereas nonintenders’ food-handling behavior did not significantly improve, *b*s < 0.21, *z*s < 2.74, *p*s > 0.17 (see Figure 3).

To compare the generic condition with the control condition, we additionally fit a model with conditions included as two dummy-coded variables with generic intervention as the comparison group. The model specified was the same as the main model with the tailored condition as the comparison group. According to the results, time significantly predicted behavior, *b* = 0.29, *t*(1469) = 5.92, *p* = 0.001, whereas the HAPA phase did not, *b* = 0.02, *t*(1469) = 0.18, *p* = 0.86. There was no significant difference between the generic and the tailored condition, *b* = −0.10, *t*(926) = −1.14, *p* = 0.25, or the generic and the control condition, *b* = 0.01, *t*(926) = 0.16, *p* = 0.86. Moreover, there were no significant time and condition interaction effects over time between the generic and the tailored condition, *b* = −0.00, *t*(1469) = −0.08, *p* = 0.93. However, there was a difference in hygienic food handling over time between the generic and the control conditions, *b* = −0.10, *t*(1469) = −2.02, *p* = 0.04. The negative gradient showed that the behavior increased less over time among participants in the control condition compared to those in the generic condition. Multiple comparisons further revealed that hygienic food handling significantly increased in the control condition from T1 to T2, *b* = 0.21, *z* = 4.23, *p* = 0.001, from T1 to T3, *b* = 0.33, *z* = 6.26, *p* = 0.001, and from T1 to T4, *b* = 0.39, *z* = 6.40, *p* = 0.001. In the generic condition, results also yielded a significant increase in hygienic food handling from T1 to T2, *b* = 0.28, *z* = 4.92, *p* = 0.001, from T1 to T3, b = 0.51, *z* = 8.04, *p* = 0.001, and from T1 to T4, *b* = 0.57, *z* = 8.10, *p* = 0.001. Furthermore, it improved between T2 and T3, *b* = 0.23, *z* = 3.63, *p* = 0.01, and between T2 and T4, *b* = 0.29, *z* = 4.21, *p* = 0.001. The three-way interactions of time, condition and HAPA phase were not significant, neither for the comparison between the generic and the tailored condition, *b* = −0.11, *t*(1469) = −1.65, *p* = 0.10, nor for the comparison between the generic and the control condition, *b* = 0.06, *t*(1469) = 0.98, *p* = 0.33.

Thus, we could partially confirm hypothesis four. The generic intervention yielded a better improvement in food handling over time than the control condition. Contrary to expectations, hygienic food handling did not remain at the same level among the control condition. Moreover, we found no superior effect of the tailored intervention to the generic intervention and declined hypothesis five. Since the slope did not differ between the tailored and the control conditions, we could also not confirm hypothesis one regarding hygienic food-handling behavior. However, in the control condition, hygienic food handling increased more among intenders than among nonintenders, whereas there was no difference between intenders and nonintenders in the tailored condition.

### 3.4. Knowledge

**Figure 4:**
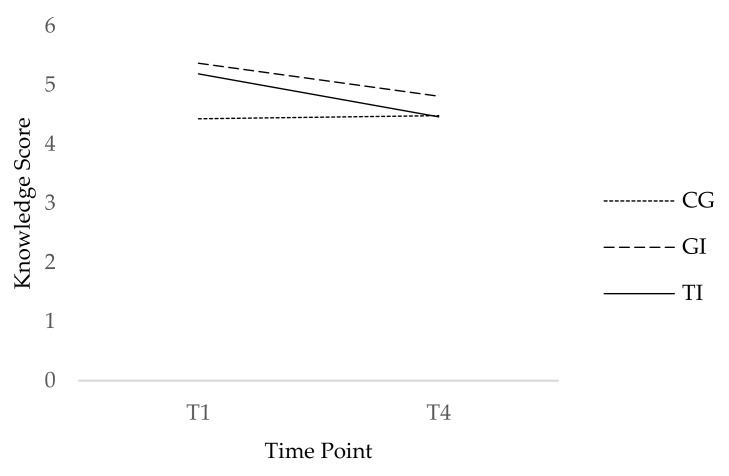
Difference in knowledge about antimicrobial resistance in food between conditions and at T1 and T4. TI denotes tailored intervention; GI: generic intervention; and CC: control condition.

The results of the repeated-measures ANOVA revealed a significant main effect of time, *F*(1, 395) = 37.56, *p* = 0.001, η^2^ = 0.09, and a significant main effect of condition on participants’ AMR knowledge, *F*(2, 395) = 12.02, *p* = 0.001, η^2^ = 0.06. Furthermore, there was a significant condition and time point interaction effect on knowledge, *F*(2, 395) = 13.94, *p* = 0.001, η^2^ = 0.07, thereby indicating that the video increased participants’ knowledge in the tailored and generic condition compared to that of the control condition at T1. However, it was a short-term effect because after two months (T4), there was no longer a difference between the three conditions (see Figure 4).

### 3.5. Evaluation

The independent *t*-test indicated that participants in the generic intervention condition (*M* = 4.67, *SD* = 0.73) evaluated the received information significantly more positively than participants in the tailored intervention condition (*M* = 3.83, *SD* = 0.50), *t*(226) = 10.16, *p* = 0.001, *d* = 1.33.

## 4. Discussion

This two-month intervention study evaluated consumers’ hygienic food handling and HAPA variables after receiving a tailored intervention compared to receiving a generic intervention or no intervention. The motivation to execute food-handling measures was used as the critical characteristic for tailoring. Therefore, the intervention strategies for participants with little intention to adopt hygienic food-handling measures were developed to address risk perception, outcome expectancy, and self-efficacy, whereas the strategies for participants with higher levels of intention should address coping planning, action control and habit formation. According to the HAPA, a change in these determinants was expected to increase nonintenders’ intention and intenders’ behavior, respectively.

However, we could not find a superior effect of the tailored intervention on food handling or the HAPA constructs compared to the generic intervention. Since participants in the generic condition evaluated the intervention more positively than participants in the tailored intervention, it is not surprising that we could not find the expected effects. In this study, the tailored intervention provided a different BCT when a participant moved from nonintender to intender, but not when a participant fell back into the non-intender phase. This approach was chosen because, according to the theory, goal setting also leads to an increase in motivation. That is, once an individual reached the intender phase, he or she only received BCTs targeting the implementation of the two selected food-handling measures. This might be why the tailored intervention was not more effective than the generic intervention. Moreover, the cut-off for reaching the intender phase was relatively strict. Only participants with the highest intention response (score 7 ‘fully intend’) to implement a certain measure received the intervention for intenders. However, it might be possible that the BCTs for intenders would also be more appropriate for participants with a lesser, but still high, intention level.

Another possible explanation is that the participants did not receive the tailored intervention at the exact time of need. So-called just-in-time adaptive interventions provide tailored support to a current need state [17]. For example, the BCTs’ coping advice might be more effective when advice on overcoming barriers is provided immediately when a barrier occurs. The same applies to the BCTs targeting the preintentional variables. Triggering self-efficacy might be more effective immediately after a person failed to perform a particular behavior. Additionally, all nonintenders in the tailored intervention received a BCT firstly to increase self-efficacy, secondly to decrease negative outcome expectancy and finally to increase risk perception. Tailoring the timing and temporal order of BCTs might increase the effects of a tailored intervention. Yet, an alternative explanation for our result may be that BCTs other than those selected would be more appropriate for a tailored intervention in this context, although we selected these BCTs based on previous research and theory. Implementing BCTs differently from those used in the original studies (e.g., by varying the content or the medium) also possibly impacted our findings because it is not specified in the literature how these techniques should be organized and applied. It is difficult to relate our results to previous studies that proved the positive impact of tailored messaging because study populations, intervention contents and behavior outcomes were considerably different. Perhaps most importantly, most other investigations were focused on one specific behavior, whereas our intervention attempted to address multiple food-handling practices. In future research, an open question about the acceptance of the received intervention at the end of the study should be added to gather information on how to improve the applied BCTs for consumers.

Although we found no differences between the tailored and the generic intervention regarding effects on hygienic food handling or HAPA variables, there were differences between the tailored and the control conditions. For the most important dependent variable, that is, hygienic food-handling behavior, the tailored intervention proved to have added benefit beyond that conferred in the control condition. The tailored intervention improved hygienic food handling for participants in both HAPA phases, whereas the information in the control condition only improved intenders’ food-handling behavior. The improvements in the condition without intervention were contrary to our hypotheses. The contents in the control condition were apparently sufficient to provide intenders with the necessary information to overcome the intention–behavior gap. Analyses indicated that respondents with a higher interest in the topic of hygienic food handling participated in our study (see Table S4). A more positive attitude towards surveys among participants may have also led to this result.

Participants who dropped out after T2 had higher levels of perceived likelihood than completers. To drop out of the study, although perceiving a personal risk of AMR through food, might be explained by repression of health concerns. The apparent low perceived likelihood among intenders can be attributed to their more frequent implementation of hygienic food-handling practices. The preventive behavior decreases their personal likelihood of AMR through food. Moreover, even if participants believed in executing the suggested hygienic food-handling behavior, this does not imply that they implemented the measures correctly. The intenders in the tailored intervention received more information on the correct implementation of hygienic food-handling measures. Therefore, it would be interesting to know whether the accuracy of the implementation was higher among intenders in the tailored condition than the other participants (i.e., intenders in the generic and control condition as well as nonintenders from all three conditions).

For the postintentional determinants (i.e., the mediators between intention and behavior), we were surprised to find that coping planning and action control increased more among nonintenders than intenders in the tailored condition. Only the intenders in the tailored intervention received a BCT to increase coping planning. The reason for this rather contradictory result is not completely clear, but it can be assumed that intenders generally experienced barriers more often as they implemented more hygienic food-handling measures. Therefore, they possibly already planned how to overcome these barriers before attending this study, whereas nonintenders did not. This finding indicates that nonintenders also perceived barriers due to improving their hygienic food-handling behavior, which prevented them from implementing new hygienic measures, and they considered how to overcome these barriers without prompting them. The same might account for the results regarding action control.

Contrary to the postintentional variables, the results of some of the preintentional determinants were as expected. Self-efficacy and likelihood of risk increased more among the participants in the tailored condition than those in the control condition. For self-efficacy, the tailored intervention yielded a superior effect for nonintenders but not for intenders. This confirms that the BCT aiming to increase self-efficacy was successful since only nonintenders received this strategy. Intenders in the tailored intervention neither received a BCT to improve self-efficacy nor raise the perceived likelihood of risk. Nevertheless, the latter increased more among participants in the tailored condition than the control condition regardless of the individual’s HAPA phase. As shown in previous research, this might be due to the educational video provided in the tailored and generic intervention at T1 [38]. The personalized BCT risk message had no additional effect on risk perception; otherwise, risk perception should have increased among nonintenders in the tailored condition compared to the participants without the risk message.

Moreover, the goal of the nonintenders’ tailored intervention was to decrease negative outcome expectancies by mitigating negative beliefs about hygienic measures, rather than to increase positive outcome expectancies (i.e., the goal of the BCT was that negative beliefs would be outweighed by existing positive beliefs). Therefore, it is not surprising that we did not find a change in positive outcome expectancies. However, the applied BCT failed to achieve the expected effect on negative outcome expectancy. Interestingly, intenders’ beliefs regarding negative consequences due to unhygienic food-handling practices increased in the tailored condition. It is probable that participants experienced some of the negative consequences due to the adoption of the hygienic food-handling measures (e.g., they are time-consuming). An intervention including goal setting to improve hygienic food handling should thus also address negative outcome expectancies.

Among the tailored condition, an increase in the preintentional determinants was expected to enhance intention among nonintenders. Although self-efficacy and perceived likelihood of risk increased more among participants in the tailored condition than in the control condition, we could not find the same result for intention. It can thus be reasonably assumed that the information about AMR provided in the control condition (i.e., the questionnaire) increased nonintenders’ intention. Apart from psychosocial predictors, other predictors can influence the intention to conduct preventive health behavior. Among the tailored and the control conditions, intention increased among nonintenders but not among intenders. This is in line with theory and was the main goal of the BCTs provided in the tailored intervention for nonintenders. The tailored intervention closed the gap between nonintenders’ and intenders’ intention, whereas a difference in intention remained between nonintenders and intenders in the control condition. Given that intention was used to define intenders and nonintenders, the result should consequently be treated with caution. Moreover, the high level of intention among intenders suggests that a ceiling effect is likely to be present—that is, the scale could only discriminate among respondents in the moderate-to-high range of behavioral intention [63].

Despite the fact that the interventions did not always yield the expected interaction effects, intenders generally showed higher scores on the relevant determinants compared to nonintenders. This is in line with theory. Thus, the perceived likelihood was the only determinant for which the intenders had a similarly low mean score as the nonintenders, but the correlation between likelihood and intention was lower than the other preintentional variables. Apart from the main effect of the HAPA phase, we found the main effects of time on behavior and intention, but also on the preintentional determinant perceived likelihood and the postintentional determinant coping planning.

The implementation of hygienic measures as a habitual behavior did not increase during the two months of the intervention study. However, this is not particularly surprising if we consider that behaviors need many repetitions in consistent settings to become a habit. Since the sample prepared raw meat several times a month to a maximum of once a week on average, two months was too short a timeframe to sufficiently repeat the behaviors. On a wider level, future research should examine under which conditions self-guided behavior change occurs to achieve a long-term behavior change. The short-term increase in knowledge after watching the video indicates that a sustainable change of a psychosocial factor cannot be generally assumed.

A key finding of the study was that merely providing brief information about the health risk of AMR (i.e., the content of the control condition) appears to be sufficient to yield an increase in some behavioral determinants and hygienic food-handling behavior. The information given by the questionnaire items may have affected participants. However, since more consumers with a low intention for hygienic food handling were excluded from analysis, it might be that this effect mostly occurred among consumers with a higher motivation to improve their food-handling behavior.

Another explanation may be that hygienic food handling should not only address the psychological determinants of behavior, but also include a physical environment that fosters the behavior, such as the close presence of soap. Since the intenders in the tailored intervention received more specific information about implementing measures (e.g., instruction including dish sponges, brushes, and dishwashing detergent), environmental barriers might were perceived more by this group. One’s environment is acknowledged by the HAPA model as a moderator of intention, planning, and action.

### Strengths and Limitations

The main strength of this intervention study was the application and testing of theory-based BCTs, which are required to ensure that effective interventions are implemented in practice. To our knowledge, this study is the first study testing a tailored intervention to improve consumers’ hygienic food-handling behavior to combat the spread of AMR through food. Moreover, the anonymous data collection promoted the accuracy and truthfulness of the study’s findings. Further, the study included a representative sample according to age and gender from the German-speaking part of Switzerland. The longitudinal design enabled us to assess behavior change within the same individuals over time. However, since the follow-up survey at T4 was one month after the last intervention, no conclusions should be drawn about the long-term effects on hygienic behavior change that extend beyond this period. Based on the rather short follow-up period, we recommend extending the follow-up time in future research to observe the long-term effects.

A limitation of our study is the retrospective self-report measure of hygienic food-handling behavior, which might be biased due to recall bias, i.e., past events being remembered inaccurately [64]. The current intervention study was further limited to consumers in Switzerland without taking into account differences among cultures and countries. Moreover, the participants were recruited from an online survey panel. We set quotas for the sample to ensure that it represented the Swiss population. However, it is possible that those who participated until the end (T4) found the topic more interesting than those who dropped out earlier.

Furthermore, dropouts at T1 differed from the rest of the sample in respect to some behavioral determinants. Participants received detailed information about the research subject during T1. If individuals had received the information during recruitment (i.e., before T1), those who dropped out might not have started the study, and the self-selection would thus have occurred before data collection. It is further important to mention that the *p*-value for age across the three intervention groups is approximately significant (see Table 1). *p*-values near 0.05 may indicate an insufficient sample size. In the case of a repeated-measures MANOVA, the results with our final sample would be slightly underpowered, which could have reduced the chance of detecting an expected small effect. However, in multilevel studies, the main problem is usually the sample size at the group level (i.e., participant level in case of a longitudinal study) because the group-level sample size is always smaller than the repeated individual-level sample size (i.e., repeated measures in case of a longitudinal study) [65]. In two-level models, an individual-level sample size of at least 100 for accurate estimation of higher levels is suggested.

## 5. Conclusions

We observed that a tailored intervention was more successful in improving the preintentional determinants of self-efficacy and perceived likelihood of risk among nonintenders than in that of postintentional determinants among intenders. Although merely presenting information about AMR in food produced positive effects on hygienic food handling on its own, providing an intervention with theory-based BCTs proved to additionally increase preintentional HAPA determinants. Therefore, tailoring concerning the intention to improve hygienic food handling appears to be promising since interventions yielded different effects for nonintenders and intenders. However, our findings also suggest that a tailored intervention is not generally more successful in promoting preventive health behavior. In the context of hygienic food-handling behavior to mitigate the spread of AMR bacteria, a generic intervention was proved to yield the same effect on behavioral determinants and hygienic food-handling behavior as a tailored intervention. Future research should adapt the tailored intervention to the changing state of the HAPA phase to investigate if a tailored intervention can achieve more hygienic food handling than a generic intervention. Moreover, more research is needed to determine whether other BCTs can replace those that had little effect in the current study.

## Figures and Tables

**Table 1:** Randomization checks of variables measured at T1 over all three conditions.

			Condition					
	Tailored(*n* = 268)		Generic(*n* = 280)		Control(*n* = 381)			
	*n*	%	*n*	%	*n*	%	χ^2^ (*df*)	*p*
*Gender*							0.43	0.81
Male	118	44.0	116	41.4	160	42.0		
Female	150	56.0	164	58.6	221	58.0		
*Education level* ^1^							2.62	0.62
Primary or secondary school	14	5.2	19	6.8	19	5.0		
Vocational or higher secondary school	156	58.2	150	53.6	225	59.1		
College/University degree	97	36.2	111	39.6	137	36.0		
*Household composition* ^1^							11.57	0.07
Single-person household	67	25.0	52	18.6	89	23.4		
With partner	77	28.7	99	35.4	139	36.5		
With children	91	34.0	108	38.6	123	32.3		
Another household composition	32	11.9	21	7.5	30	7.9		
	***M***	***(SD)***	***M***	***(SD)***	***M***	***(SD)***	***F* (df)**	***p***
Age	43.52	(14.27)	43.80	(13.52)	45.90	(13.50)	3.01 (2, 926)	0.05
*Frequency of preparing…*								
Raw red meat	3.60	(1.08)	3.67	(1.05)	3.64	(1.14)	0.26 (2, 926)	0.77
Raw poultry	3.36	(1.10)	3.26	(1.12)	3.39	(1.16)	1.03 (2, 926)	0.36
Raw fish/seafood	2.49	(1.09)	2.35	(1.21)	2.50	(1.23)	1.52 (2, 926)	0.22

*Note:*^1^ missing value for one participant.

**Table 2:** Results of linear multilevel analyses with the preintentional determinants as dependent variable.

	Self-Efficacy		Likelihood		Severity		Positive Outcome Expectancy		Negative Outcome Expectancy	
	γ	95% CI	γ	95% CI	γ	95% CI	γ	95% CI	γ	95% CI
*Fixed effects level 1*										
Intercept	5.55	5.41; 5.69	3.12	2.94; 3.29	4.77	4.58; 4.96	5.67	5.51; 5.83	3.14	2.98; 3.31
Time	0.18	−0.01; 0.36	0.18	0.07; 0.29	−0.12	−0.28; 0.03	0.06	−0.20; 0.31	−0.08	−0.17; 0.01
*Fixed effects level 2*										
Control condition ^#^	0.18	−0.00; 0.37	0.17	−0.05; 0.40	0.05	−0.20; 0.30	0.06	−0.15; 0.26	−0.06	−0.27; 0.16
Generic condition ^#^	0.10	−0.10; 0.30	0.24	−0.01; 0.48	0.16	−0.11; 0.43	0.08	−0.14; 0.30	−0.07	−0.30; 0.16
HAPA phase ^†^	0.60	0.41; 0.79	0.21	−0.03; 0.44	0.40	0.13; 0.67	0.47	0.24; 0.70	−0.37	−0.59; −0.16
*Cross-level interaction*										
Time * control condition	−0.13	−0.23; −0.02	−0.20	−0.34; −0.06	−0.01	−0.16; 0.14	−0.12	−0.24; 0.01	0.11	−0.00; 0.22
Time * generic condition	0.07	−0.04; 0.17	−0.06	−0.21; 0.08	0.09	−0.06; 0.25	−0.02	−0.15; 0.11	0.06	−0.05; 0.18
Time * HAPA phase * control condition	0.21	0.07; 0.34	0.11	−0.07; 0.29	0.05	−0.14; 0.25	0.13	−0.04; 0.30	−0.26	−0.42; −0.11
Time * HAPA phase * generic condition	−0.02	−0.16; 0.13	0.04	−0.15; 0.23	−0.09	−0.30; 0.12	0.01	−0.08; 0.27	−0.26	−0.41; −0.10
*Random effect variances*										
Intercept	0.72		0.92		0.94		0.57		0.91	
Time	0.02		0.05							
Residuals	0.32		0.57		0.88		0.65		0.48	
Corr ^§^	−0.14		−0.12							
AIC	6034.87		7358.03		7867.48		6996.59		6746.83	

*Notes.* γ denotes the regression coefficient. ^#^ Tailored intervention is the comparison group. ^†^ HAPA phase: Intender coded as 1, nonintender coded as 0. * denotes an interaction between two or three variables. For the hypothesis, only relevant interactions are reported. **^§^** Correlation of the intercept and slope.

**Table 3:** Results of linear multilevel analysis with intention, postintentional determinants, and self-reported hygienic food-handling behavior as dependent variables.

	Intention		Coping Planing		Action Control		Habit		Behavior	
	γ	95% CI	γ	95% CI	γ	95% CI	γ	95% CI	γ	95% CI
*Fixed effects level 1*										
Intercept	5.06	4.92; 5.20	4.01	3.80; 4.23	4.62	4.41; 4.84	5.10	4.92; 5.29	4.39	4.27; 4.51
Time	0.63	0.43; 0.82	0.57	0.23; 0.92	−0.07	−0.37 0.21	0.07	−0.15; 0.30	0.28	0.19; 0.38
*Fixed effects level 2*										
Control condition ^#^	0.11	−0.07; 0.30	0.18	−0.10; 0.46	0.08	−0.20; 0.36	0.06	−0.17; 0.30	0.11	−0.04; 0.27
Generic condition ^#^	0.06	−0.14; 0.26	0.21	−0.09; 0.52	0.17	−0.13; 0.48	0.08	−0.17; 0.34	0.10	−0.07; 0.27
HAPA phase ^†^	1.29	1.10; 1.48	0.74	0.43; 1.04	0.64	0.35; 0.93	0.42	0.18; 0.65	0.19	0.02; 0.35
*Cross-level interactions*										
Time * control condition	−0.09	−0.19; 0.02	−0.09	−0.27; 0.09	−0.12	−0.26; 0.05	−0.02	−0.13; 0.10	−0.09	−0.19; 0.00
Time * generic condition	0.09	−0.02; 0.20	−0.02	−0.22; 0.17	0.02	−0.14; 0.20	0.02	−0.10; 0.15	0.00	−0.10; 0.11
Time * HAPA phase * control condition	0.14	0.00; 0.27	0.24	0.00; 0.48	0.23	0.01; 0.42	0.11	−0.04; 0.27	0.17	0.05; 0.30
Time * HAPA phase * generic condition	−0.03	−0.17; 0.11	0.24	−0.01; 0.50	0.15	−0.09; 0.36	0.08	−0.09; 0.24	0.11	−0.02; 0.24
*Random effect variances*										
Intercept	0.64		1.20		1.39		1.27		0.42	
Time	0.01		0.05		0.00		0.03		0.01	
Residuals	0.37		1.09		0.95		0.46		0.30	
Corr ^§^	−0.19		0.13		−0.15		−0.29		0.55	
AIC	6132.07		8568.97		8116.87		7024.09		5454.27	

*Notes.* γ denotes the regression coefficient. ^#^ Tailored intervention is the comparison group. ^†^ HAPA phase: intender coded as 1, nonintender coded as 0. * denotes an interaction between two or three variables. For the hypothesis, only relevant interactions are reported. ^§^ Correlation of the intercept and slope.

## Data Availability

The data presented in this study are available on request from the corresponding author. The data are not publicly available because another publication is planned by the authors on a different part of the dataset.

## Supplementary Materials

The video was developed by the research team and is available online at https://doi.org/10.26041/fhnw-3409, 30 January 2022). The following are available online at https://www.mdpi.com/article/10.3390/ejihpe12020014/s1: Items and their descriptive statistics, analyses comparing dropouts, definition of antibiotic-resistant bacteria used in the control condition and illustrations used in the generic and tailored interventions.
