# Supplementary material for: Tailoring Can Improve Consumers’ Hygienic Food-Handling Behavior to Reduce the Transmission Risk of Antimicrobial-Resistant Bacteria through Food
Source: Eur J Investig Health Psychol Educ. 2022 Feb 1;12(2):169–92. doi: 10.3390/ejihpe12020014 (PMC8871282; doi:10.3390/ejihpe12020014)
Supplement: Supplementary file 1 [file ejihpe-12-00014-s001.zip › ejihpe-1475785-supplementary.pdf]

European Journal of
Investigation in Health, Psychology and Education

MDPI

Supplementary Materials

Table S1. Items and descriptive statistics of the HAPA variables.

	Items per variable	*M*	*SD*
	Behaviour		
B1	Using a separate cutting board and knife for the preparation of raw meat, poultry, fish and seafood, and a separate cutting board and knife for other foods (e.g. vegetables).	4.05	1.79
B2	Not touching or placing a smartphone on the work surface during the preparation of raw meat, poultry, fish and seafood.	4.16	1.86
B3	Washing the dish sponge and dishwashing brush thoroughly with soap and hot water after cleaning kitchen utensils used to prepare raw meat, poultry, fish or seafood.	4.36	1.53
B4	Changing kitchen towels, rags and sponges once a week and washing them at 60°C	4.92	1.28
B5	Cleaning the work surface thoroughly after preparing raw meat, poultry, fish and seafood.	5.26	0.99
B6	Defrosting frozen meat, poultry, fish or seafood in the refrigerator instead of at room temperature.	4.07	1.41
	Intention: I intend in the future…		
I1	to use a separate cutting board and knife for the preparation of raw meat, poultry, fish and seafood, and a separate cutting board and knife for other foods (e.g. vegetables).	4.74	1.95
I2	not to touch or place a smartphone on the work surface during the preparation of raw meat, poultry, fish and seafood.	5.57	1.80
I3	to wash the dish sponge and dishwashing brush thoroughly with soap and hot water after cleaning kitchen utensils.	5.66	1.51
I4	to change kitchen towels, rags and sponges once a week and wash them at 60°C	5.63	1.57
I5	to clean the work surface thoroughly after preparing raw meat, poultry, fish and seafood.	6.01	1.29
I6	to defrost frozen meat, poultry, fish or seafood in the refrigerator instead of at room temperature.	5.53	1.56
	Risk perception likelihood: How likely do you think it is that…		
RP1	food in Switzerland is contaminated with antibiotic-resistant bacteria?	4.04	1.51
RP2	you or your housemates spread antibiotic-resistant bacteria?	3.12	1.53
RP3	you or your housemates get sick because of antibiotic-resistant bacteria in food?	2.89	1.45
	Risk perception severity: How dangerous do you think it is when…		
RP4	food in Switzerland is contaminated with antibiotic-resistant bacteria?	5.13	1.41
RP5	you or your housemates spread antibiotic-resistant bacteria?	4.98	1.51
RP6	you or your housemates get sick because of antibiotic-resistant bacteria in food?	5.00	1.62
	Positive outcome expectancy		
PO1	By adopting safe food-handling measures, I expect to kill all pathogenic bacteria in food.	5.38	1.58
PO2	By implementing hygiene measures, I expect to reduce the spread of antibiotic resistance through food.	5.90	1.21
PO3	By adopting safe food-handling measures, I expect to prevent bacterial infections.	5.84	1.20
	Negative outcome expectancy		
NO1	It is exaggerated because of the hygiene standards in Switzerland, when I pay attention to safe food handling.	2.75	1.59
NO2	When I implement hygiene measures in the kitchen, it is too elaborative.	2.74	1.59

NO3	When I adopt many safe food-handling measures, then I also kill useful bacteria.	2.89	1.74
NO4	When I pay attention to hygienic food handling in the kitchen, my immune system weakens because a certain amount of germs is necessary.	3.49	1.71
NO5	When I adopt safe food-handling measures, I cannot prevent the spread of antibiotic resistance because hospitals are responsible for the spread of antibiotic resistance.	2.53	1.63
	Self-efficacy: I am confident that…		
SE1	I can regularly implement hygienic food measures if I want to.	6.17	1.13
SE2	I can adopt the hygiene measures that I do not yet implement in the future, if I want to.	6.00	1.17
SE3	I can implement these hygiene measures if I want to, even if I do not notice any direct positive consequences.	5.86	1.30
SE4	I can implement these hygiene measures if I want to, even when I am with friends or relatives who find it unnecessary.	5.78	1.30
SE5	I can implement these hygiene measures if I want to, even if it takes time until it becomes a habit.	5.79	1.25
SE6	I can regularly implement hygienic food measures, even if I do not have much time.	5.54	1.46
	Coping planning: I have specific plans for what I can do...		
CP1	if something comes up during the implementation of these hygiene measures.	3.94	1.78
CP2	when I have to overcome habits while preparing raw meat, raw poultry, raw fish or raw seafood.	4.50	1.73
CP3	when I don't have enough time to implement the hygiene measures.	4.23	1.79
CP4	when my friends or family consider these hygiene measures unnecessary.	4.64	1.85
	Action control		
AC1	I always observe whether I implement hygienic food-handling measures.	4.84	1.69
AC2	I pay attention to which hygienic food-handling measures I implement.	4.94	1.59
	Habitual behaviour: Implementing hygienic food-handling measures is something...		
H1	I do automatically.	5.44	1.43
H2	I have no need to think about doing.	5.33	1.45
H3	I do without having to consciously remember it.	5.26	1.47
H4	I start to do before I am aware that I am doing it.	5.05	1.58

Note. Behaviour items were assessed on 6-point response scales, ranging from 1 = 'never' to 6 = 'always'. Pre- and post-intentional variables were assessed on 7-point response scales, with higher scores indicating higher agreement with the item.

Table S2. Knowledge items and response frequencies.

		correct<br>*n* (%)	incorrect<br>*n* (%)
K1	Antibiotic resistance means that bacteria are able to resist the effects of antibiotics.	864 (93.0)	65 (7.0)
K2	Only people with a weakened immune system need to protect themselves from antibiotic-resistant bacteria.*	846 (91.1)	83 (8.9)
K3	If a person suffers from food poisoning (e.g. caused by infected raw chicken), he/she may carry antibiotic-resistant bacteria.	624 (67.2)	305 (32.8)
K4	Antibiotic-resistant bacteria can be transmitted from animals to humans.	749 (80.6)	180 (19.4)
K5	People always get sick when they carry antibiotic-resistant bacteria.*	646 (69.5)	283 (30.5)
K6	There is no risk of antibiotic-resistant bacteria in organic foods.*	757 (81.5)	172 (18.5)

Notes. The response scale for the knowledge items was ‘true’, ‘false’, and ‘I don’t know’. Incorrect and ‘I don’t know’ answers were merged together. *Item is incorrect.

S1. Definition of antibiotic resistant bacteria

Definition of antibiotic resistant bacteria shown to participants in the control condition

Antibiotic-resistant bacteria can be transmitted to humans and animals through various exposure pathways. Food can also be contaminated with resistant bacteria. If antibiotic-resistant bacteria cause an infection, the infection is difficult or impossible to treat with existing antibiotics. Simple hygiene measures during food preparation help to reduce the risk of transmission and the spread of resistant bacteria.

S2. Illustrations BCT, shown in the generic intervention

We would like to remind you to implement hygiene measures during food preparation. Hygienic food handling contributes to mitigating the spread of antibiotic-resistant bacteria. Proper washing, separating, cooking and chilling are important basic rules when handling food.

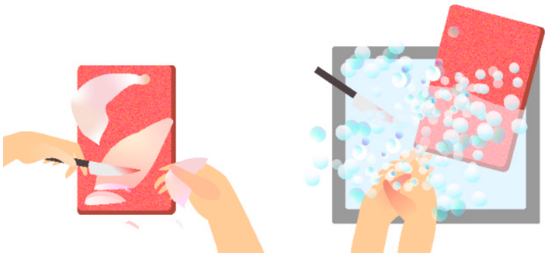

1) Wash hands and kitchen utensils (e.g. cutting board or dishwashing brush) immediately after contact with raw meat, poultry, fish and seafood thoroughly with warm water and soap.

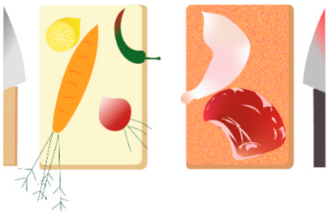

2) Avoid contact of raw meat, poultry, fish and seafood with other foods by chilling and preparing them separately.

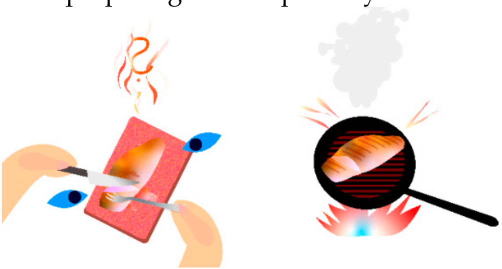

3) Make sure that the meat, poultry, fish and seafood are well heated and cooked to a safe internal temperature (at least 70°C).

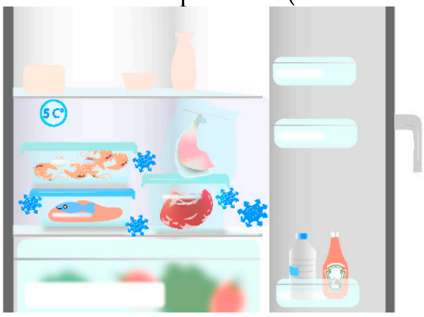

4) Store meat, poultry, fish and seafood in the refrigerator at 5°C.

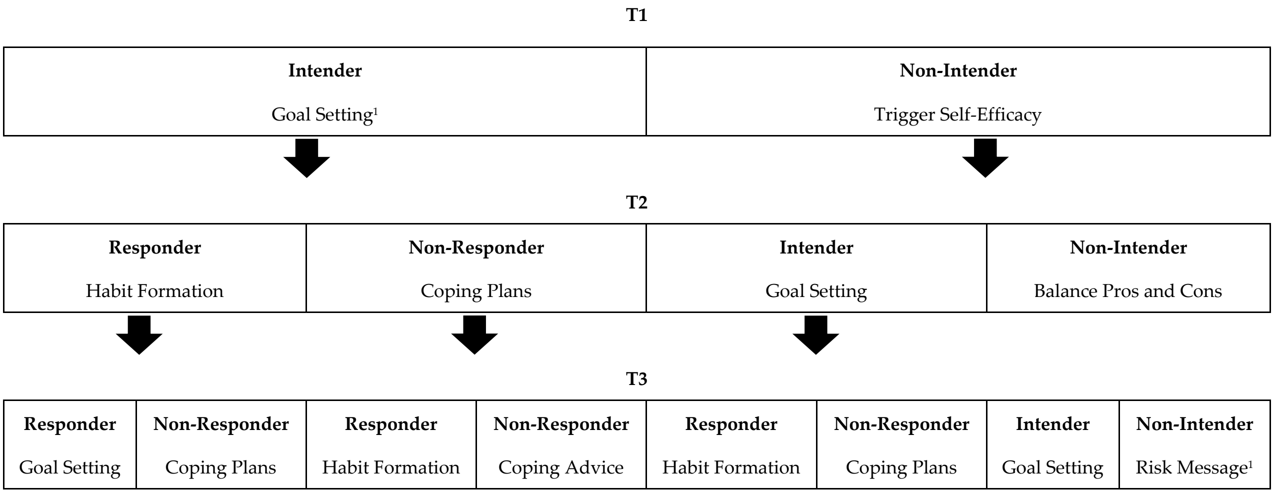

Figure S1. Overview of the behaviour change strategies (BCTs) targeting different HAPA variables used in the tailored intervention condition.

S3. Illustrations in the tailored intervention

Goal setting BCT: Advices including an illustration and a short text

The following illustrations were provided in the tailored intervention as advice corresponding to their chosen goal. Corresponding to the goals they set, each respondent received two illustrations with explanations.

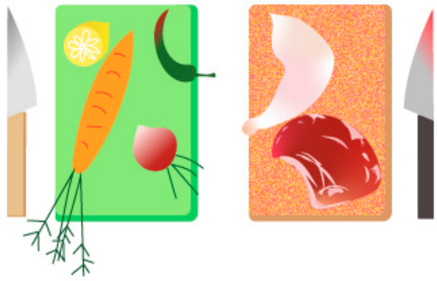

1)

Use cutting boards of different colours: one cutting board for fruit and vegetables and another cutting board for the raw animal products (meat, poultry, fish or seafood) you are preparing. Inform your family members or housemates which colour is used for which food.

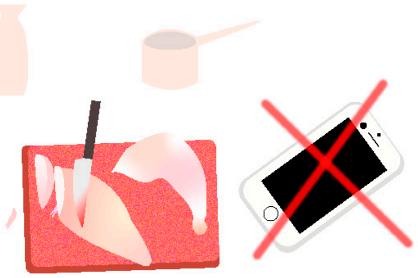

2)

Smartphones are crawling with bacteria. In order to prevent antibiotic-resistant bacteria on your display, it is important to place your smartphone outside the kitchen during the preparation of raw poultry, raw meat, raw fish or raw seafood. Wash your hands thoroughly with soap and warm water before touching your smartphone after preparing food.

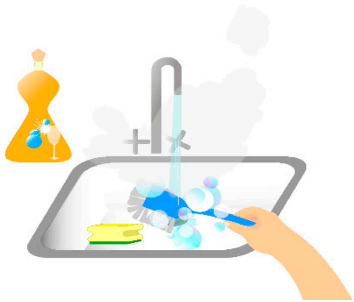

3)

Dish sponges and brushes are a bacteria hotbed. Antibiotic-resistant bacteria can also land on dish sponges and dishwashing brushes and then be further spread in your kitchen. After washing kitchen utensils used to prepare raw meat, poultry or fish, rinse sponges and brushes thoroughly with hot water (for at least 20 seconds). Let the sponges dry completely afterwards. Place the dishwashing

detergent next to the sink to prevent touching anything before washing up. Dishwasher-safe washing brushes can be washed in the dishwasher's cutlery basket.

4)
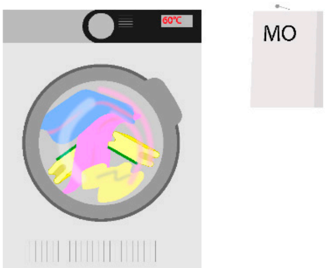

Select one day of the week on which you will change used kitchen towels and sponges. Wash these at a temperature of 60°C. A hot wash will kill possible antibiotic-resistant bacteria.

5)
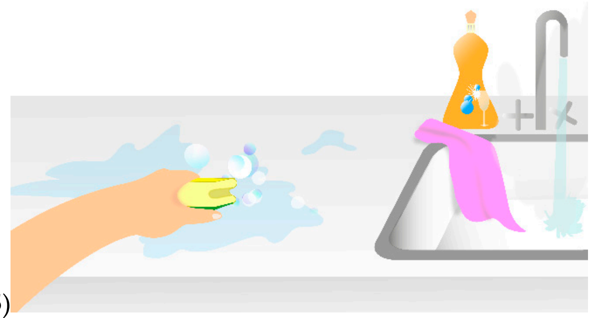

Wash both the kitchen utensils and the surface immediately after the preparation of raw meat. Use warm water and dishwashing detergent to remove possible bacteria and to prevent transmission to other food. Then dry the washed kitchen surface because bacteria love moisture. Wash the kitchen sponge after cleaning the surface thoroughly to avoid bacteria spreading in the kitchen. The refrigerator should also be cleaned every four weeks.

6)
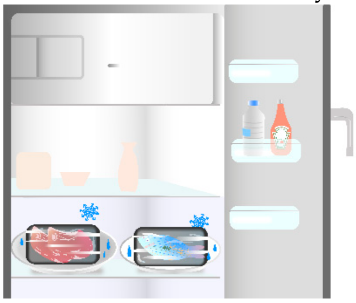

Store the meat with the food packaging on a high plate or in a bowl in the refrigerator because unpacked meat promotes the spread of bacteria. Take the meat out of the freezer the evening before. If you do not have the time to do this, you can defrost the meat in a water bath. Place the packaged meat in warm water. Change the water when it has cooled down. Thawing frozen meat at room temperature is neither efficient nor safe. It becomes susceptible to bacteria, which are particularly active at room temperature. Defrosted meat should always be prepared immediately and not frozen a second time.

Risk Message BCT: Pictograph and Tailored Text Message

You prepared poultry meat [Tailored Content] during the past two weeks. Random samples have shown that up to 45% of poultry meat (i.e. 4.5 out of 10 chicken legs) are contaminated with antibiotic-resistant Campylobacter bacteria (see the figure below).

Campylobacter bacteria cause gastrointestinal diseases. Antibiotic-resistant Campylobacter bacteria prolong the antibiotic treatment of the disease.

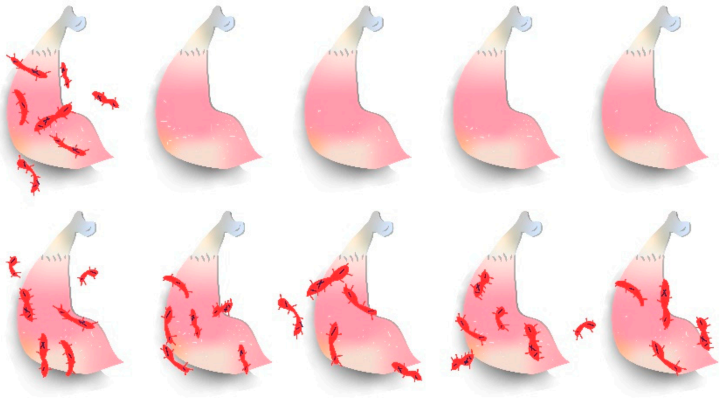

We have taken poultry meat as an example because it is a popular meat in Switzerland. It accounts for a quarter of all meat consumption. Other meat and food are also contaminated with antibiotic-resistant bacteria. Therefore, you also have a risk of transmitting antibiotic-resistant bacteria if you prepare meats other than poultry. By implementing the hygiene measures, you can enjoy the meat without any concerns!

Table S3. Analyses comparing dropouts at T1, T2 and T3 with completers from the final data sample on demographics and HAPA variables assessed at T1.

	Dropouts T1	Dropouts T2	Dropouts T3	Completers T4			
	n = 315	n = 96	n = 120	n = 398			
	n (%)	n (%)	n (%)	n (%)	χ^2 (df)	p	
Gender					6.62 (3)	.09	
Male	125 (39.7)	32 (33.3)	56 (46.7)	181 (45.5)			
Female	190 (60.3)	64 (66.7)	64 (53.3)	217 (54.5)			
Education level[1]					10.31 (6)	.11	
Primary or secondary school	20 (6.3)	4 (4.2)	9 (7.5)	19(4.8)			
Vocational or higher secondary school	190 (60.3)	45 (46.9)	73 (60.8)	223 (56.0)			
College/University degree	105 (33.3)	46 (47.9)	38 (31.7)	156 (39.2)			
Household composition					25.61 (9)	.002	
Single-person household	62 (19.7)^A	12 (12.5)^A	27 (22.5)	107^B (26.9)			
With partner	105 (33.3)	25 (26.0)	45 (37.5)	140 (35.2)			
With children	117 (37.1)	42 (43.8)	37 (30.8)	126 (31.7)			
Another household composition	31 (9.8)	16 (16.7)	11 (9.2)	25(6.3)			
	M (SD)	M (SD)	M (SD)	M (SD)	F (df)	p	η^2_p
Age	42.95 (13.83)^A	39.17 (12.20)^A	44.97 (14.64)	47.06 (13.26)^B	11.13 (3, 925)	.001	0.04
HAPA variables							
Behaviour	4.44 (0.82)	4.37 (0.81)	4.42 (0.85)	4.53 (0.86)	1.40 (3, 925)	.24	
Intention	5.53 (1.34)	5.67 (1.30)	5.38 (1.30)	5.69 (1.15)	2.43 (3, 925)	.06	
Risk perception: Likelihood	3.27 (1.24)	3.74 (1.32)^A	3.26 (1.24)	3.35 (1.21)	3.82 (3, 925)^B	.01	0.01
Risk perception: Severity	4.95 (1.45)	5.00 (1.21)	5.09 (1.41)	5.10 (1.39)	0.75 (3, 925)	.53	
Self-efficacy	5.78 (1.14)	5.90 (0.96)	5.84 (1.13)	5.91 (1.07)	0.87 (3, 925)	.46	
Positive outcome expectancy	5.73 (1.11)	5.74 (0.94)	5.78 (1.17)	5.66 (1.17)	0.44 (3, 925)	.73	
Negative outcome expectancy	3.11 (1.21)	3.06 (1.23)	3.15 (1.24)	2.91 (1.16)	2.19 (3, 925)	.09	
Coping planning	4.26 (1.57)	4.44 (1.43)	4.23(1.69)	4.38 (1.50)	0.70 (3, 925)	.55	
Action control	4.78 (1.56)	5.11 (1.40)	4.80 (1.68)	4.95 (1.58)	1.52 (3, 925)	.21	
Habit	5.18 (1.39)	5.43 (1.20)	5.25 (1.48)	5.31 (1.32)	1.04 (3, 925)	.37	

Notes. Completers include participants who completed all four questionnaires. Values with different superscripts per variable indicate that the values differ significantly from the completers ($p < .05$).

Table S4. Analyses comparing dropouts between participants excluded between T1 and T4 and those analysed.

	Excluded n = 256	Analysed n = 929			
	n (%)	n (%)	χ^2 (df)	p	
Gender[1]			0.42 (1)	.52	
Male	85 (45.0)	394 (42.4)			
Female	104 (55.0)	535 (57.6)			
Education level[1]			3.45 (2)	.18	
Primary or secondary school	4 (4.9)	52 (5.6)			
Vocational or higher secondary school	39 (47.6)	531 (57.2)			
College/University degree	39 (47.6)	345 (37.1)			
Household composition[1]			1.17 (3)	.76	
Single-person household	20 (24.4)	208 (22.4)			
With partner	29 (35.4)	315 (33.9)			
With children	24 (29.3)	322 (34.7)			
Another household composition	9 (11.0)	83 (8.9)			
	M (SD)	*M (SD)*	t (df)	p	d
Age	40.52 (16.12)	44.58 (13.76)	-3.30 (1,126)	.01	0.27
HAPA variables					
Behaviour	4.39 (0.84)	4.47 (0.84)	-1.07 (1,078)	.28	
Intention	5.26 (1.51)	5.59 (1.26)	-2.52 (1,071)	.01	0.24
Risk perception: Likelihood	3.89 (1.43)	3.35 (1.24)	2.60 (1,063)	.01	0.40
Risk perception: Severity	4.81 (1.54)	5.04 (1.39)	-1.73 (1,060)	.08	
Self-efficacy	5.45 (1.36)	5.86 (1.09)	-3.22 (1,055)	.00	0.33
Positive outcome expectancy	5.48 (1.47)	5.87 (1.10)	-2.88 (1,047)	.01	0.30
Negative outcome expectancy	3.78 (1.65)	3.03 (1.20)	4.70 (1,039)	.00	0.52
Coping planning	4.52 (1.52)	4.33 (1.54)	1.26 (1,036)	.21	
Action control	4.91 (1.53)	4.89 (1.57)	0.17 (1,033)	.87	
Habit	5.11 (1.47)	5.27 (1.35)	-1.07 (1,021)	.29	

Notes. [1]This variable includes missing values, as participants already dropped out before demographics were assessed.
